# Cell-specific pattern of berberine pleiotropic effects on different human cell lines

**DOI:** 10.1038/s41598-018-28952-3

**Published:** 2018-07-13

**Authors:** Alessandro Agnarelli, Marco Natali, Mercedes Garcia-Gil, Rossana Pesi, Maria Grazia Tozzi, Chiara Ippolito, Nunzia Bernardini, Robert Vignali, Renata Batistoni, Anna Maria Bianucci, Silvia Marracci

**Affiliations:** 1 Department of Biology, University of Pisa, Pisa, Italy; 2 Department of Clinical and Experimental Medicine, University of Pisa, Pisa, Italy; 3 Interdepartmental Research Center “Nutraceuticals and Food for Health”, University of Pisa, Pisa, Italy; 4 Istituto Nazionale per la Scienza e Tecnologia dei Materiali, Florence, Italy

## Abstract

The natural alkaloid berberine has several pharmacological properties and recently received attention as a potential anticancer agent. In this work, we investigated the molecular mechanisms underlying the anti-tumor effect of berberine on glioblastoma U343 and pancreatic carcinoma MIA PaCa-2 cells. Human dermal fibroblasts (HDF) were used as non-cancer cells. We show that berberine differentially affects cell viability, displaying a higher cytotoxicity on the two cancer cell lines than on HDF. Berberine also affects cell cycle progression, senescence, caspase-3 activity, autophagy and migration in a cell-specific manner. In particular, in HDF it induces cell cycle arrest in G2 and senescence, but not autophagy; in the U343 cells, berberine leads to cell cycle arrest in G2 and induces both senescence and autophagy; in MIA PaCa-2 cells, the alkaloid induces arrest in G1, senescence, autophagy, it increases caspase-3 activity and impairs migration/invasion. As demonstrated by decreased citrate synthase activity, the three cell lines show mitochondrial dysfunction following berberine exposure. Finally, we observed that berberine modulates the expression profile of genes involved in different pathways of tumorigenesis in a cell line-specific manner. These findings have valuable implications for understanding the complex functional interactions between berberine and specific cell types.

## Introduction

Tumorigenesis is a multi-step process depending on modifications of multiple cell signaling pathways. During tumor progression cancer cells acquire genetic and epigenetic changes that cause functional heterogeneity, with important implications for cancer therapy. When a pathway is blocked in a tumor cell, because of an anti-tumor treatment, other pathways can be in fact activated allowing the cell to evade the inhibition. For these reasons, the use of phytochemicals with multi-targeting properties and relatively low toxicity may be an interesting approach for implementing cancer therapy^1,2^. Moreover, the use of natural compounds may reduce the deleterious side effects exerted on non-tumor cells by chemotherapics^2^.

The natural alkaloid berberine is a multi-targeting compound with several pharmacological properties, including anti-tumor activity^2^. Berberine may affect different molecular targets depending on the cell type^3^. For example, it impairs mitochondrial function and triggers the release of pro-apoptotic factors into the cytosol^2–4^ leading to activation of caspases, but can also activate non-apoptotic pathways of cell death^4,5^. It has also been reported that berberine induces senescence^6^ in U251 and U87 glioblastoma cells. The ability of inducing senescence as well as alternative cell death pathways is an interesting feature of berberine that can be potentially used for arresting the growth or killing cancer cells that fail to die by apoptosis^6–9^. Furthermore, berberine can inhibit the signaling pathways of cell migration and invasion that are key processes in metastatic progression^10^. Recent studies indicate that berberine may also modulate epigenetic patterns^11,12^ whose changes may be of relevance in cancerogenesis^13^.

In this work, we studied how berberine affects cell cycle progression, senescence, autophagy and migration in two human tumor cell lines, U343 glioblastoma cells and MIA PaCa-2 pancreatic adenocarcinoma cells, using HDF as a non-tumor control. To give an insight into the molecular targets by which berberine affects tumorigenesis, we also analyzed the expression profile of several genes affecting cancer progression.

## Results

### Intracellular localization of berberine

**Figure 1:**
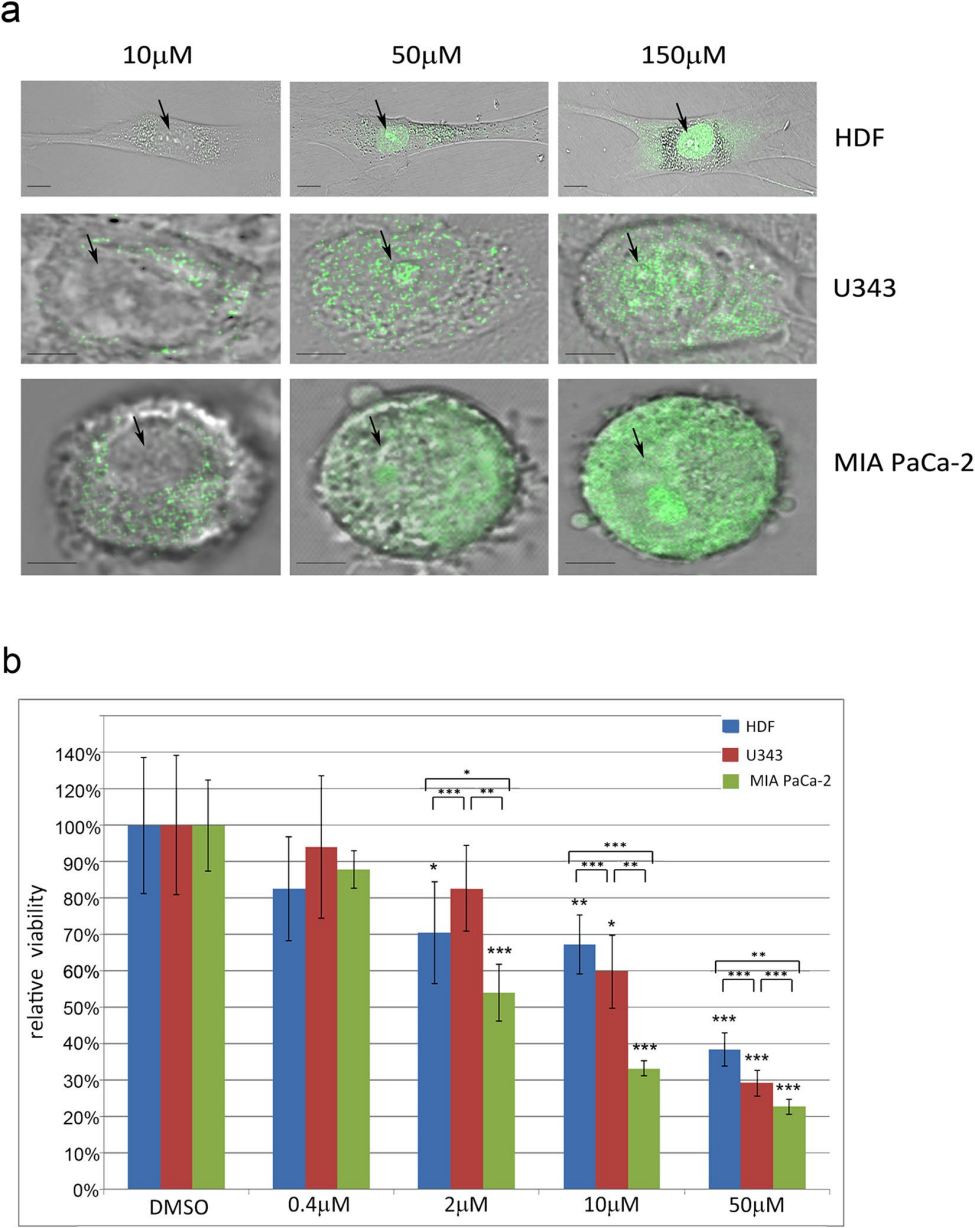
Intracellular localization of berberine and effects on viability in HDF, U343 and MIA PaCa-2 cells. (**a**) Confocal images of berberine distribution in HDF, U343 and MIA PaCa-2 cells. Cells were photographed 1 hour after treatment with berberine (10 μM, 50 μM or 150 μM). Black arrows point out nuclei. Scale bars represent 5 μm. (**b**) Reduction of cell viability after 48 hours of treatments with 0.4 μM, 2 μM, 10 μM, 50 μM berberine in HDF, U343 and MIA PaCa-2 cells. Graph columns represent mean of viable cells ± S.D. normalized versus control group (DMSO). *P < 0.05; **P < 0.01; ***P < 0.001.

**Figure 2:**
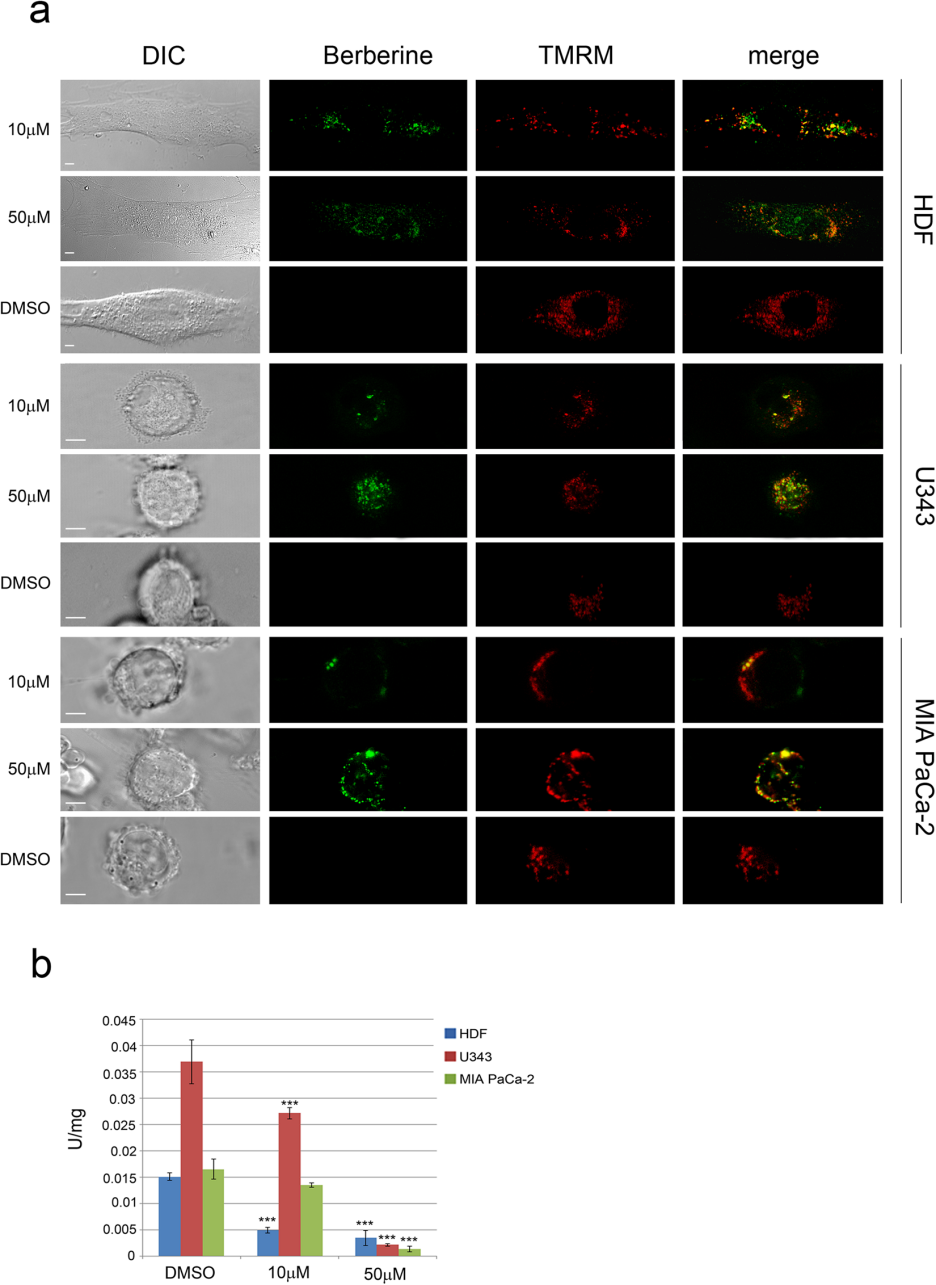
Berberine localizes in mitochondria and affects mitochondrial function. (**a**) Berberine was visualized by confocal microscopy in mitochondria of HDF, U343 and MIA PaCa-2 cells after 48 hours of exposure to 10 μM or 50 μM berberine. Merge columns represent overlapping of the berberine green signal with the TMRM red signal. DMSO-treated cells, used as a control, lack green fluorescence. Differential interference contrast (DIC) highlighted the cell morphology. Scale bars indicate 5 μm. (**b**) Citrate synthase activity was measured in the three cell lines after treatments in the presence or absence of berberine as described in Methods. U = Units of enzymatic activity. *P < 0.05; **P < 0.01; ***P < 0.001.

Berberine emits light-green fluorescence when excited by the 488 nm laser line. By confocal microscopy we have analyzed the intracellular localization of berberine in HDF, U343 and MIA PaCa-2 cells, treated for 1 hour with different concentrations of this alkaloid (Fig. 1a). We observed that at 10 µM concentration, berberine is mainly distributed in the cytoplasm. The fluorescent signal appears weaker in HDF than in U343 and MIA PaCa-2 cells (Fig. 1a). At higher berberine concentrations (50 μM or 150 μM), the signal is clearly visualized both in cytoplasm and nucleus (Fig. 1a). This localization is maintained also after 48 hours of berberine exposure (Fig. 2a). Control cells that received the vehicle dimethyl sulfoxide (DMSO) alone did not display any fluorescence signal.

### Berberine decreases cell viability

To analyze how different concentrations of berberine affect cell viability, we performed cell counting by using the trypan blue dye exclusion method. As shown in Fig. 1b, when HDF, U343 and MIA PaCa-2 cells were treated with a low dose of berberine (0.4 μM), no significant alteration of cell viability was observed, indicating that this concentration does not induce cell death. Treatments with 2 μM, 10 μM or 50 μM berberine for 48 hours reduced viability in MIA PaCa-2 cells (54%, 33%, 23% viability, respectively), as well as in HDF (70%, 67%, 38% viability, respectively). Conversely, while cell viability did not significantly change after treatment with 2 μM berberine, the exposure to higher doses (10 μM or 50 μM) significantly reduced viability in U343 cells (60%, 29% viability, respectively). It may be interesting that after treatments with 10 μM or 50 μM berberine, cell viability declined more in the tumor cell lines than in non-tumor HDF (p < 0.001, p < 0.01, respectively for U343 and MIA PaCa-2 cells compared to HDF).

### Berberine localizes in mitochondria and impairs mitochondrial function

The use of the mitochondrial tracer tetramethylrhodamine methyl ester (TMRM)^14^ provides evidence that berberine can also localize in the mitochondria of HDF, U343 and MIA PaCa-2 cells, as revealed by the partial overlapping of the berberine green signal with the TMRM red signal (Fig. 2a). In addition, citrate synthase activity was measured as an index of mitochondrial function. Citrate synthase localizes in the mitochondrial matrix and catalyzes the first reaction of the tricarboxylic acid cycle. Citrate synthase activity was found to be similar in untreated HDF and MIA PaCa-2 cells, while U343 cells exhibited two-fold activity compared to the other cell lines (Fig. 2b). Upon treatment with 10 μM berberine for 48 hours, this activity was significantly reduced (p < 0.001), relative to controls, to 32.7% in HDF and to 73.5% in U343 cells, but not in MIA PaCa-2 cells. The treatment with 50 μM berberine further reduced citrate synthase activity in HDF and U343 (to 23.1% and 5.8%, respectively) and also showed a significant effect on MIA PaCa-2 cells (reduction to 8.5%). Altogether these results suggest that berberine induces a mitochondrial dysfunction in the three cell types.

### Berberine alters cell cycle progression and induces senescence in HDF, U343 and MIA PaCa-2 cells

**Figure 3:**
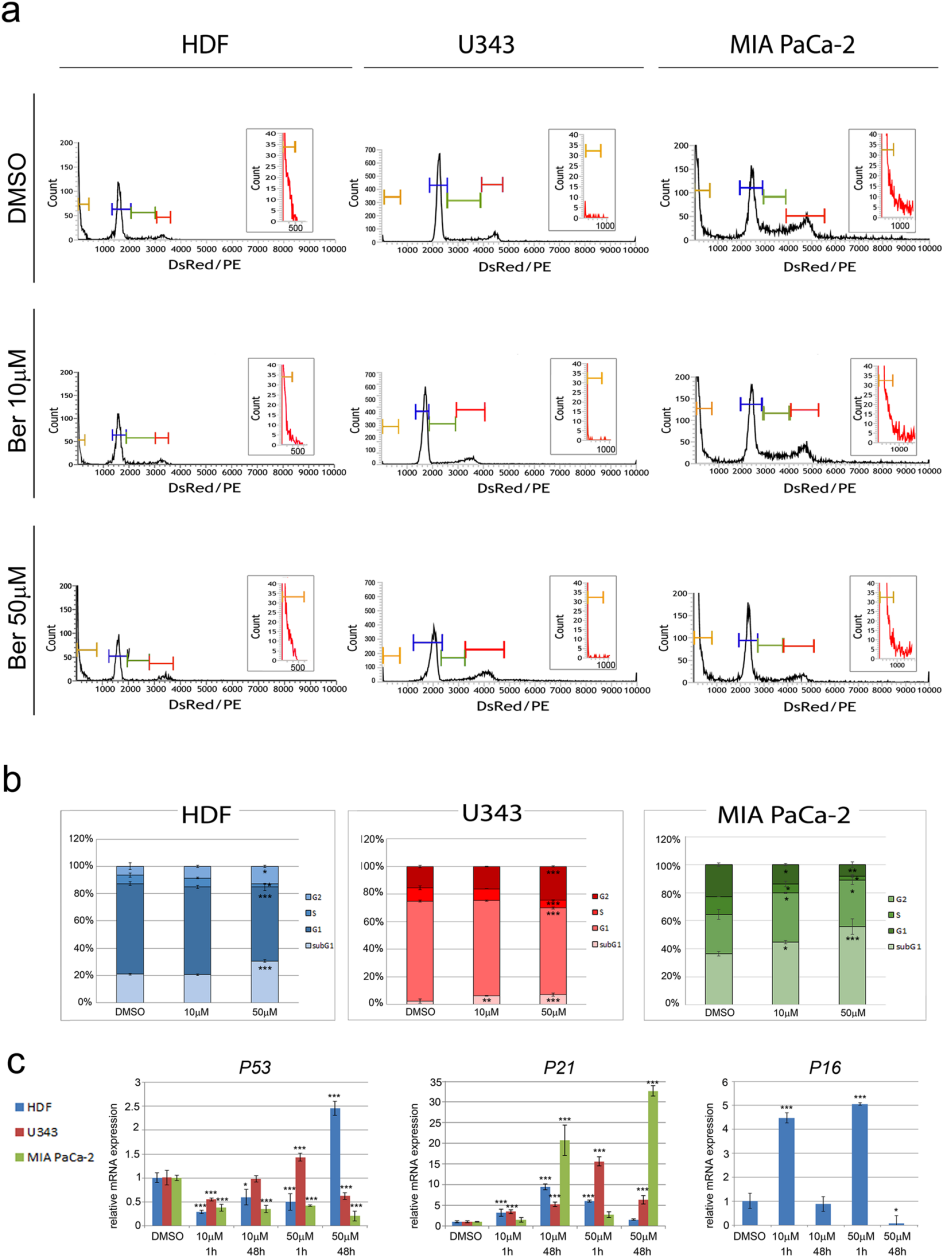
Berberine alters the cell cycle and the transcriptional profile of cell cycle regulators. (**a**) Representative images of cell cycle distribution of HDF, U343 and MIA PaCa-2 cells. Cell cycle distribution was assayed by flow cytometry, following treatment with 10 μM or 50 μM berberine for 48 hours; subG1 (yellow bars), G1 (blue bars), S (green bars), G2 (red bars). Upper right insets represent the zoomed in subG1 population relative to each graph. Cell number (count) is reported in y-axis; fluorescence intensity (DsRed/PE) is reported in x-axis. (**b**) Quantification of cell cycle distribution; (**c**) The transcriptional profile of *P53*, *P21*, *P16* was analyzed by Real Time RT-PCR after 1 hour or 48 hours berberine treatments. *P < 0.05; **P < 0.01; ***P < 0.001.

We next examined berberine effects on cell cycle progression. A lower dose (10 μM) of the alkaloid was sufficient to induce G1 arrest in MIA PaCa-2 cells after 48 hours, but did not alter the cell cycle of HDF and U343 cells (Fig. 3a,b). Figure 3a,b also shows that 10 μM berberine increased the percentage of U343 and MIA PaCa-2 cells found in subG1 whereas no effect was detected in HDF. In particular, the subG1 population of cells treated with vehicle was 20.9%, 2.5% and 36.5% in HDF, U343 and MIA PaCa-2 respectively and increased to 6.3% in U343 and 44.8% in MIA PaCa-2 cells following treatment with 10 μM berberine. In addition, treatment with 50 μM berberine for 48 hours induced G2/M arrest in HDF and U343 cells and G1 arrest in MIA PaCa-2 cells (Fig. 3a,b). In the three cell lines, this treatment also caused a significant decrease of S phase cells. 50 μM berberine also raised the percentage of cells found in subG1 in the three cell lines relative to untreated cells, and again the highest effect was found in MIA PaCa-2 cells (55.8% compared to 30.6% in HDF and 7.1% in U343 cells).

In addition we analyzed the transcription profile of important regulators of cell cycle, such as *Tumor Protein P53 (P53), Cyclin Dependent Kinase Inhibitor 2 A (P16) and Cyclin Dependent Kinase Inhibitor 1 A (P21)* (Fig. 3c). Our results demonstrate that in HDF, *P53* was upregulated after treatment with 50 μM berberine for 48 hours (2.4-fold compared to untreated cells), while downregulation (to 0.25-fold) was detected at earlier times (1 hour) and also with 10 μM berberine both for 1 or 48 hours. In U343 cells, *P53* was upregulated after treatment with 50 μM berberine for 1 hour (1.4-fold) while it was downregulated (about 0.5-fold) following 10 μM berberine for 1 hour or 50 μM berberine for 48 hours; no change in the expression level was detected after incubation with 10 μM berberine (48 hours) in these cells. It is known that MIA PaCa-2 cells express a mutated form of *P53*^15^; we observed that *P53* was always downregulated (to about 0.24-fold) in these cells after berberine treatments.

In HDF, berberine modulated the expression profile of *P16* in a time- and dose-dependent manner. *P16* upregulation was observed after 1 hour of incubation with 10 μM or 50 μM berberine (4.5-fold and 5-fold, respectively). *P16* downregulation (to 0.1-fold) was found after treatment with 50 μM berberine for 48 hours. No change was detected after exposure to 10 μM berberine for 48 hours (Fig. 3c). This gene is upregulated in many tumor cells^16^, but it is not expressed in MIA PaCa-2 and U343 cells^15,17^. We did not observe any activation of *P16* transcription in these cells following berberine treatments.

In HDF, *P21* was upregulated (2.5-fold to 9-fold) after different berberine incubations excluding 50 μM for 48 hours that did not induce any alteration (Fig. 3c). *P21* transcription was also increased in U343 cells following all berberine treatments and especially in MIA PaCa-2 cells after 48 hours (20-fold to 33-fold).

**Figure 4:**
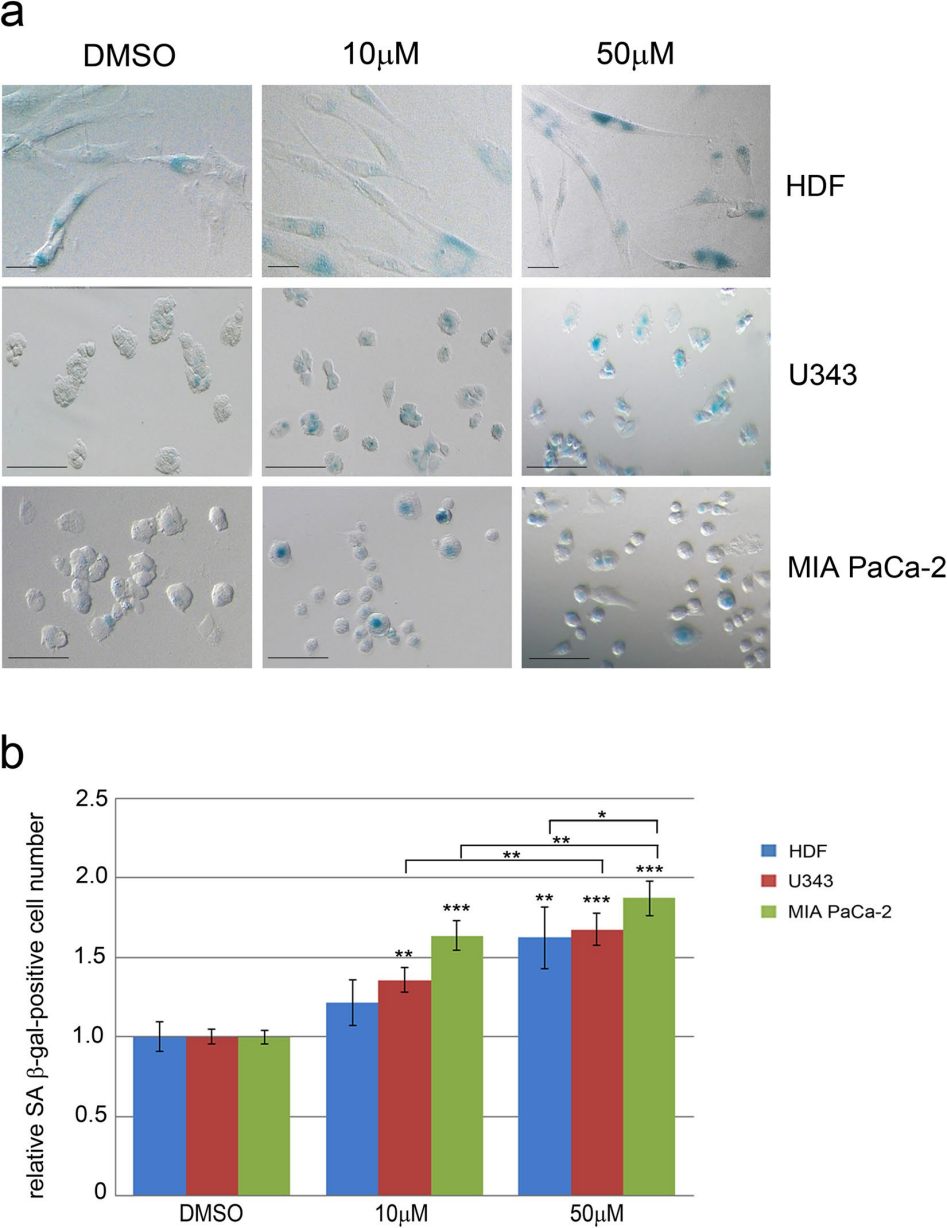
Berberine induces cell senescence in HDF, U343 and MIA PaCa-2 cells. (**a**) Brightfield images of SA β-gal-positive HDF, U343 and MIA PaCa-2 cells after berberine treatments. Treatments: 10 μM or 50 μM berberine or DMSO for 48 hours. Scale bars indicate 50μm. (**b**) The graph shows the number of SA β-gal-positive cells normalized to the control (DMSO). *P < 0.05; **P < 0.01; ***P < 0.001.

Since P21 is also a hallmark of cellular senescence we investigated the effect of berberine on senescence by evaluating the senescence-associated β-galactosidase (SA β-gal) staining (Fig. 4). Incubation for 48 hours caused a dose-dependent increase of the number of SA β-gal-positive cells in the three cell lines. In particular, 10 μM or 50 μM berberine promoted senescence in U343 and MIA PaCa-2 cells, while only the later treatment induced senescence in HDF. The senescence increase caused by 50 μM berberine was higher in MIA PaCa-2 cells than in HDF (Fig. 4a,b).

### Caspase-3 activity after treatment with berberine

**Figure 5:**
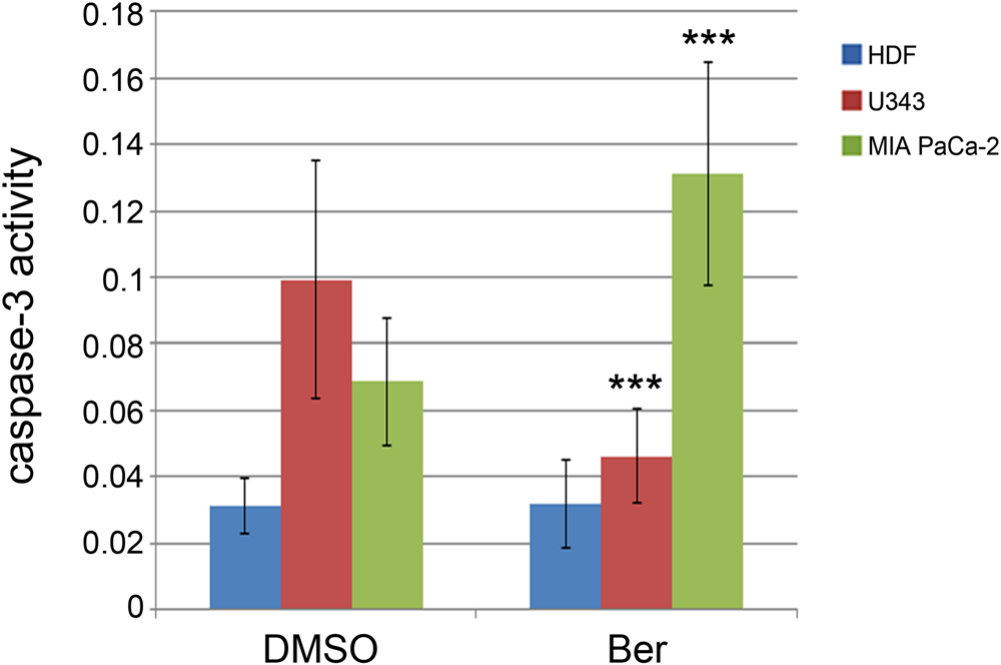
Effects of berberine on caspase-3 activity. (**a**) HDF, U343 and MIA PaCa-2 cells were incubated for 48 hours with 50 μM berberine and analyzed for caspase-3 activity monitored for 5 hours. Caspase-3 activity was measured as absorbance variation/hour/mg protein. Ber = berberine. *P < 0.05; **P < 0.01; ***P < 0.001.

To assess if berberine had a pro-apoptotic action in the analyzed cell types, we evaluated caspase-3 activity after incubation with 50 μM berberine for 48 hours. This treatment reduced to 46.5% the enzymatic activity in U343 cells when compared to the control. The same treatment increased two-fold caspase-3 activity in MIA PaCa-2 cells (Fig. 5) and did not induce any change of activity in HDF.

### Berberine increases autophagy in U343 and MIA PaCa-2 cells, but not in HDF

**Figure 6:**
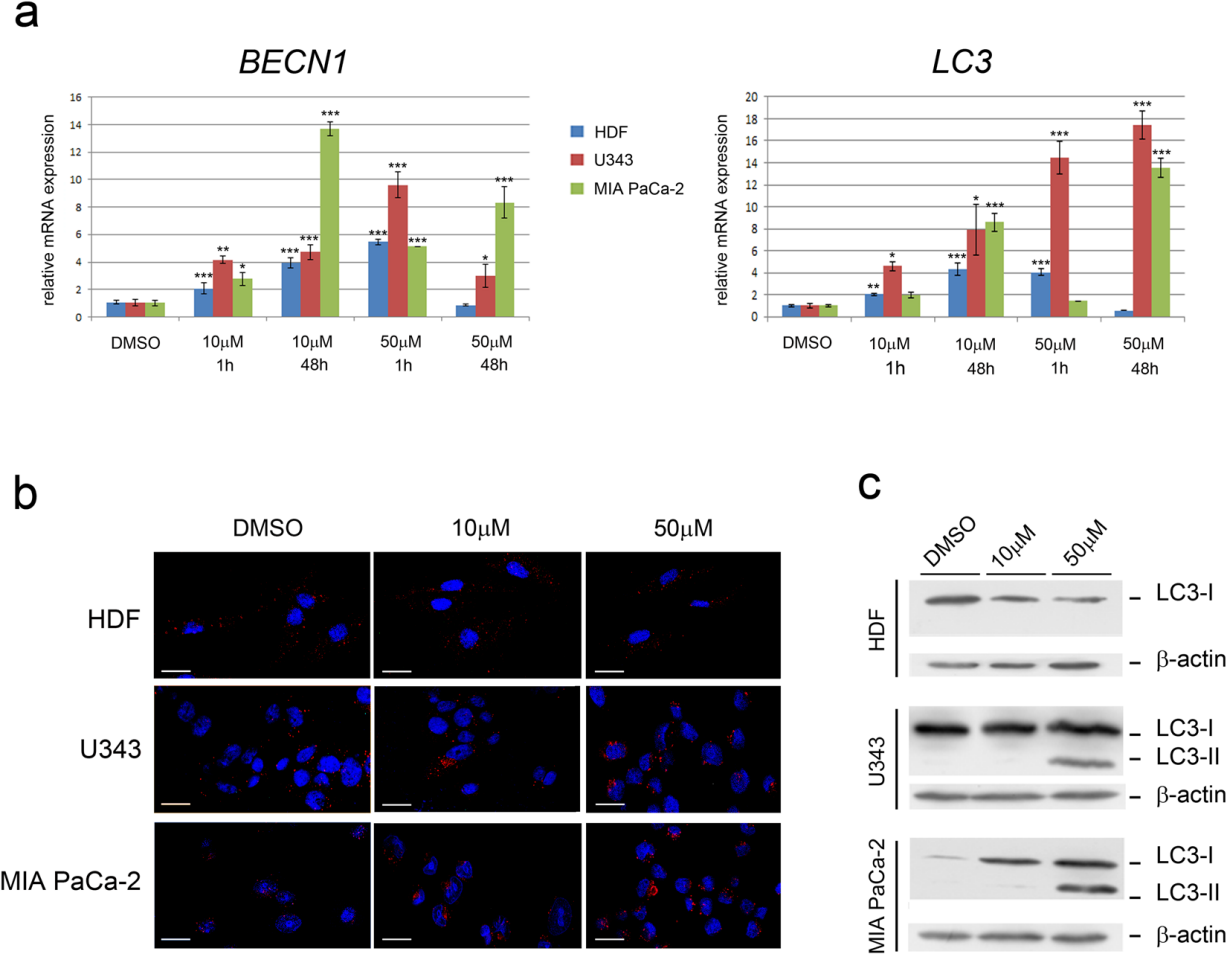
Berberine induces autophagy in U343 and MIA PaCa-2 cells, but not in HDF. (**a**) Relative mRNA expression is reported in y-axis. The treatments with berberine or DMSO alone are in x-axis. The values reported in graphs represent the mean ± S.D. (**b**) Immunofluorescence experiments. Cells were treated with berberine or DMSO for 48 hours and immunohistochemistry was performed with LC3 antibody (red fluorescence), as described in Methods. Nuclei are visualized by Hoechst staining. (**c**) Cells were treated as in (**b**) and proteins were subjected to electrophoresis and western blotting as described in Methods. *P < 0.05; **P < 0.01; ***P < 0.001.

In another series of experiments, we evaluated the expression profile of genes involved in autophagy: *Beclin 1 (BECN1), Microtubule-associated protein 1 light chain 3 beta (LC3)* and *Death-Associated Protein 1 (DAP1)*^18,19^. In HDF, 10 μM berberine for 1 or 48 hours, or 50 μM berberine for 1 hour upregulated *BECN1* expression (2-fold to 5-fold). *BECN1* transcription level was also increased by berberine in U343 and MIA PaCa-2 cells in all experimental conditions tested. Highest expression of *BECN1* was found in MIA PaCa-2 cells after 48 hours (Fig. 6a). The treatment with 10 μM berberine for 1 or 48 hours and 50 μM berberine treatment for 1 hour induced *LC3* upregulation (up to 5-fold) in HDF. *LC3* transcription level was also highly increased in U343 cells (5-fold to 18-fold) and in MIA PaCa-2 cells (8-fold to 14-fold) after 48 hours of treatments (Fig. 6a).

We investigated whether the changes in *LC3* transcription were also associated with modifications in protein expression levels by using immunofluorescence and western blot assays (Fig. 6b,c). Immunofluorescence experiments showed presence of LC3 in the three cell lines. After 48 hours of berberine treatment (10 μM or 50 μM) the fluorescent signal increased in U343 cells (50 μM) and in MIA PaCa-2 cells, but not in HDF (Fig. 6b). Western blot analysis showed presence of the autophagosome-associated LC3-II form both in U343 and MIA PaCa-2 cells treated with 50 μM berberine for 48 hours (Fig. 6c and Fig. SI1). Only the cytosolic LC3-I form was observed in untreated or 10 μM berberine-treated HDF and U343 cells (Fig. 6c and Fig. SI1). These results demonstrate that 50 μM berberine induces autophagosome formation in U343 and MIA PaCa-2 cells, but not in HDF.

### Berberine reduces migration and invasion capability in MIA PaCa-2 cells

Finally, to check the possible effects of berberine on the migratory and invasive abilities of cell lines, we performed wound healing and trans-well invasion assays.

**Figure 7:**
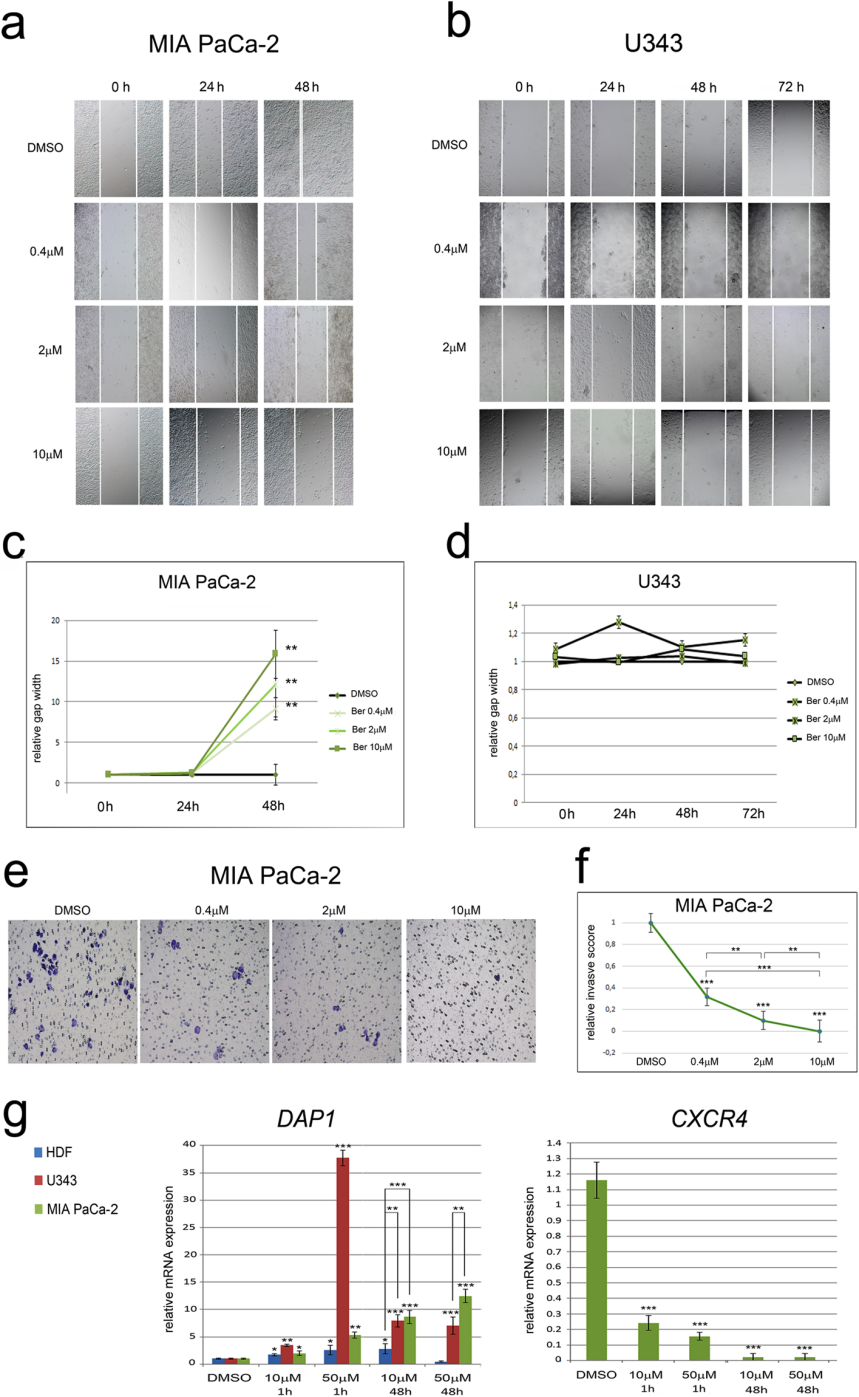
Wound healing and cell invasion assays. (**a**) Representative brightfield images of the scratches (marked by white lines) in MIA PaCa-2 cells at 0, 24 and 48 hours, after treatments with berberine or DMSO. (**b**) Representative brightfield images of the scratches in U343 cells at 0, 24, 48 and 72 hours, after treatments with berberine or DMSO. (**c**,**d**) The values reported in the graphs represent the mean distance taken at each time from the wound edges (normalized to the DMSO group) ±S.D. Ber = berberine. (**e**) Impaired invasion of berberine-treated MIA PaCa-2 in transwell assay. (**f**) The graph reports the relative invasive score (±S.D.) corresponding to each berberine concentration, compared to DMSO. (**g**) The transcriptional profile of *DAP1* and *CXCR4* was analyzed by Real Time RT-PCR. The treatments with berberine or DMSO alone are in x-axis. The values reported in graphs represent the mean ± S.D. *P < 0.05; **P < 0.01; ***P < 0.001.

Wound healing assay showed that berberine treatment (0.4 μM, 2 μM, 10 μM) for 48 hours inhibited migration (Fig. 7a,c) and invasion capability (Fig. 7e,f) of MIA PaCa-2 cells. No reduction of migration capability was observed in MIA PaCa-2 cells following exposure to different concentrations of berberine for 24 hours. In U343 cells wound healing was not observed even after 72 hours of DMSO or berberine treatment (Fig. 7b,d).

We then analyzed the transcription profile of *DAP1*, a gene involved in apoptosis, autophagy and migration^19^ and of *C-X-C Motif Chemokine Receptor 4 (CXCR4)* involved in cell migration^20^.

*DAP1* expression was upregulated in the two tumor cell lines, following berberine treatments, with the highest expression level observed in U343 after exposure to 50 μM berberine for 1 hour (37-fold). In HDF, the expression level of *DAP1* was upregulated (to 3-fold) following treatments for 1 hour with 10 μM or 50 μM berberine, as well as with 10 μM berberine for 48 hours (Fig. 7g).

The berberine treatments drastically downregulated *CXCR4* (0.22 to 0.01-fold) in MIA PaCa-2 cells; on the other hand *CXCR4* was not expressed at all in either treated or untreated U343 (Fig. 7g).

These results suggest that berberine limits the migration and invasiveness capacity of MIA PaCa-2 cells, but does not modify the behavior of U343 cells.

### Berberine alters the transcription profile of DNA methyl transferases *DNMT1*, *DNMT3A*, *DNMT3B* and *MGMT*

**Figure 8:**
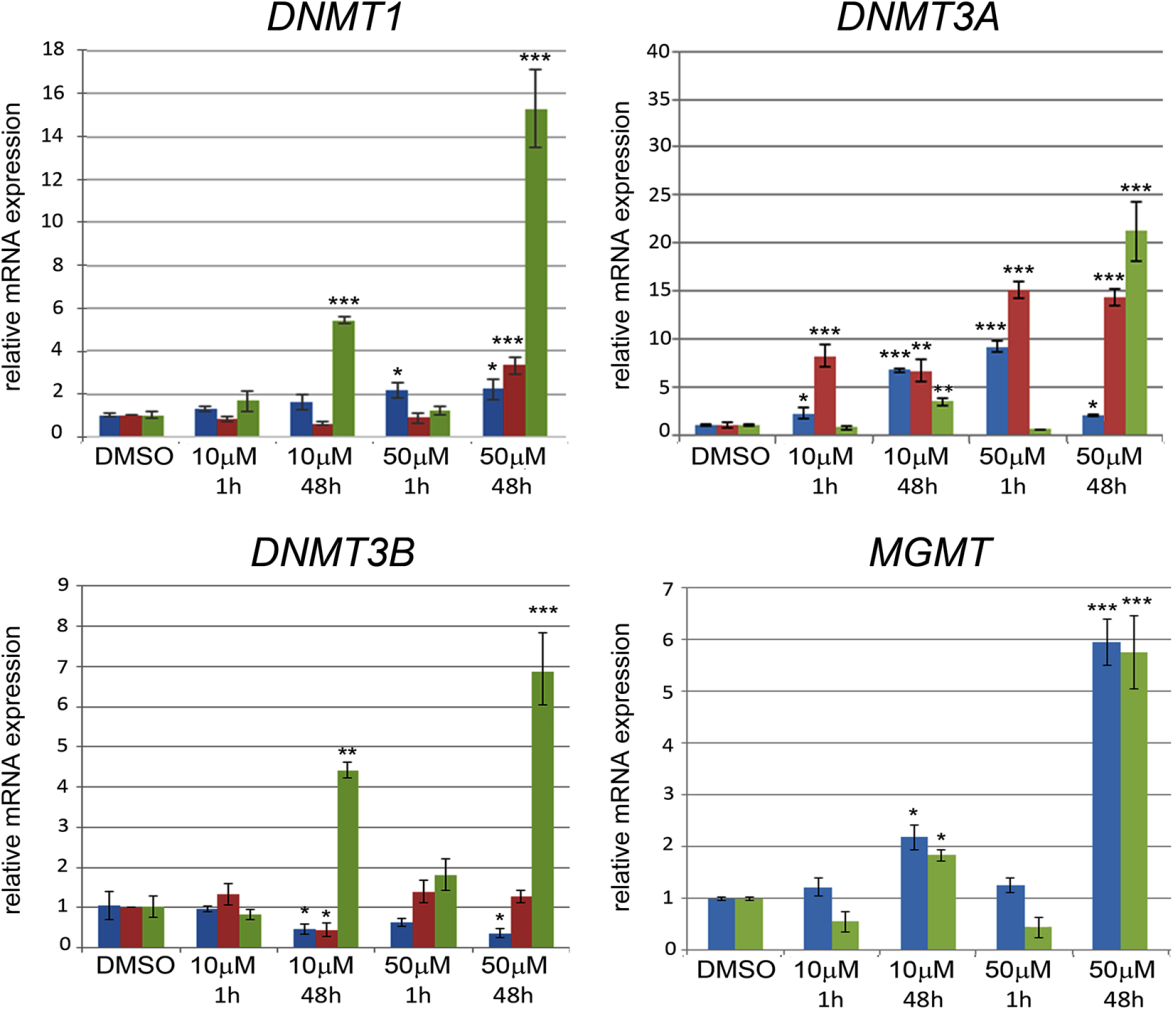
Effects of berberine on the transcriptional profile of *DNMT1, DNMT3A, DNMT3B* and *MGMT* in HDF, U343 and MIA PaCa-2 cells. Cells were treated with berberine for 1 hour or 48 hours and Real Time RT-PCR was performed as described in Methods. The treatments with berberine or DMSO alone are in x-axis. The values reported in graphs represent the mean ± S.D. *P < 0.05; **P < 0.01; ***P < 0.001.

An important aspect of tumor progression is the regulation of expression of DNA methyltransferases involved in epigenetic control and of enzymes implicated in DNA repair mechanisms^21^. We therefore analyzed the effect of berberine treatments on the transcription profile of the epigenetic regulators *DNA (cytosine-5) methyltransferase 1 (DNMT1), DNA (cytosine-5) methyltransferase 3A (DNMT3A), DNA (cytosine-5) methyltransferase 3B (DNMT3B)*, and of *O6-methylguanine DNA methyltransferase (MGMT)* involved in DNA repair (Fig. 8). HDF showed a slight increase (2-fold) in *DNMT1* transcripts level only when exposed to 50 μM berberine for 1 or 48 hours (Fig. 8). *DNMT1* was also upregulated in U343 cells (4-fold) after 50 μM berberine for 48 hours and in MIA PaCa-2 cells after treatment with both 10 μM and 50 μM berberine for 48 hours (5-fold and 15-fold, respectively).

*DNMT3A* transcription was upregulated in HDF treated with 10 or 50 μM berberine for 1 hour (2.5-fold and 9-fold, respectively) and for 10 μM berberine for 48 hours (6-fold). Berberine increased *DNMT3A* transcription in U343 cells both at 1 and 48 hours (7-fold to 15-fold) and in MIA PaCa-2 cells at 48 hours (3-fold and 23-fold with 10 or 50 μM berberine, respectively) (Fig. 8).

*DNMT3B* transcription was downregulated (up to 0.5-fold) after 48 hours of incubation with berberine both in HDF (10 or 50 μM) and in U343 cells (10 μM), while it was upregulated in MIA PaCa-2 cells (4.2-fold to 6.9-fold) (Fig. 8).

*MGMT* was upregulated in both HDF and MIA PaCa-2 cells after 48 hours of berberine treatments (2-fold to 6-fold) but not in U343 cells (Fig. 8).

These data demonstrate that berberine alters the expression level of *DNMT1, DNMT3A, DNMT3B* and *MGMT* in a cell line-specific manner.

## Discussion

The multi-targeting properties of naturally-occurring bioactive compounds are promising in cancer therapy^2^. In the present study, we have investigated the multiple effects of berberine on HDF, U343 and MIA PaCa-2 cells. In particular, we have addressed its impact on cell viability, mitochondrial function, cell cycle progression, senescence, caspase-3 activity, autophagy and migration/invasion abilities. We also analyzed berberine effects on the transcriptional profile of different molecular targets including epigenetic and DNA repair regulators.

Berberine decreases the number of viable HDF, U343 and MIA PaCa-2 cells, with different cytotoxic effects in the three cell types. Particularly, we found that 10 μM and 50 μM concentrations of berberine exert a higher cytotoxic effect in U343 and MIA PaCa-2 cells with respect to the non-tumor HDF. Our results are consistent with viability data obtained in other tumor cell lines, suggesting that cancerous cells are more sensitive to certain doses of berberine than non-tumor cells^2,3^.

As the decrease in viability observed in the three cell lines could be due to a decrease in proliferation and/or an increase in cell death and senescence, we first analyzed the effect of berberine on cell cycle. The transcriptional profile of some key players in the regulation of cell cycle and/or senescence, such as *P53*, *P21* and *P16*, was also analyzed^16,22–24^. Senescence is considered a permanent cell cycle arrest in G1 phase triggered by telomere shortening in aged cells. DNA damage response also causes senescence, resulting in the accumulation of the cdk inhibitors P21 and P16, thereby preventing DNA replication. It is well known that P53 regulates the expression of P21, an inhibitor of both the cyclin-dependent G1 kinases and the G2/M-specific cdc2 kinase. Therefore, P53 may be able to control both the G1 and the G2/M checkpoints^22^. However, the induction of P21 is also mediated by P53-independent mechanisms^25^ and is essential for the onset of both G1 and G2 cell cycle arrest in DNA damage response and cell senescence^26^. Berberine induces arrest of cell cycle progression at G0/G1 phase in MIA PaCa-2 cells (at both 10 μM and 50 μM concentrations) and at G2/M phase in both HDF and U343 cells (only at 50 μM), which can be related to the increased transcription of *P21*. In U343 cells the increase of *P21* induced by 50 μM berberine (1 hour treatment) could be dependent on *P53* upregulation. However, *P53* is downregulated in HDF and MIA PaCa-2 cells after treatments with 10 μM berberine, and in U343 cells treated with 50 μM berberine for 48 hours, suggesting that the *P21* increase in transcription observed in the same conditions, could be *P53*-independent. In further support of this, it is known that *P53* has a missense mutation in MIA PaCa-2 cells^15^. In any case, in most of the tested experimental conditions, berberine increases *P21* transcription, without a corresponding increase in *P53* transcription. These results suggest that the observed arrest in cycle progression may occur in a *P21*-dependent and *P53*-independent manner.

On the other hand, P16, which specifically targets Cdk4/6 and prevents their association with D-type cyclins and does not intervene in G1 arrest, is upregulated after induction of P21 and plays a key role in senescence maintenance^22^. Senescence can also be induced after G2 arrest^22^. We found that a high dose (50 μM) of berberine promotes senescence in the three cell lines, while a low dose (10 μM) enhances senescence only in the two tumor cell lines. This suggests that HDF are less sensitive to this alkaloid. Berberine effect on senescence and cell cycle progression in HDF, U343 and MIA PaCa-2 cells could be mediated by the increase in *P21* transcription above described. In addition, berberine-induced senescence could involve a *P16*-dependent pathway in HDF, which show increase in *P16* transcription, but not in U343 or MIA PaCa-2 cells which do not express this gene^15,17^, even in the presence of berberine. Berberine is known to promote senescence also in U87 and U251 human glioblastoma cells through a cell cycle arrest at a different phase (G0/G1)^6^, whereas it decreases senescence in human pulmonary non‐small cell lung carcinoma A549^27^. Senescence may also occur independently of *P16* by activation of epigenetic mechanisms^28^. The role of berberine as epigenetic regulator is starting to emerge^11,12,29^ and it has been associated with its anti-proliferative action^12^. We have found that berberine modulates the expression level of *DNMT1*, *DNMT3A, DNMT3B* mRNAs confirming the role of berberine as a possible epigenetic regulator. We also analyzed DNA methyltransferase *MGMT* that repairs alterations induced by alkylating agents^21,30^. Our results show that in HDF and in MIA PaCa-2 cells *MGMT* is upregulated after treatments with berberine for 48 hours, suggesting that these cells activate DNA repair mechanisms in response to a possible genotoxic damage induced by the alkaloid, that we showed it is able to enter the nucleus at high concentration. Consistent with this, it is known that berberine induces DNA damage in Rev3 deficient chicken B lymphocytes clones^2^, in human MG-63 osteosarcoma^31^ and in HeLa cells^32^. In U343 glioblastoma cells, the *MGMT* promoter is methylated and *MGMT* is not expressed^33^; we here confirm that *MGMT* is not transcribed in control cells and we demonstrate that it remains untranscribed in berberine-treated U343 cells. Interestingly, *MGMT* silencing, which prevents DNA repair, is found associated with a better prognosis in patients with glioblastoma, thus allowing a better response to radiation and chemotherapeutic drugs that may cause DNA damage^34^. In addition to induction of cell cycle arrest and senescence, berberine causes cell death. In fact, berberine (50 μM) increases the subG1 population in the three cell lines, while 10 μM berberine does not alter it in HDF and increases it in the tumor cells, at a modest extent in U343 cells and to a higher extent in MIA PaCa-2 cells.

In some models^2,3^, the anticancer activities of berberine comprise induction of the mitochondrial pathway of apoptosis. Depending on its concentration, berberine is localized in cytosol, mitochondria and nucleus of HDF, U343 and MIA PaCa-2 cells. Only at higher concentrations berberine is able to reach the nucleus, whereas lower concentrations are sufficient for its entrance to mitochondria leading to their dysfunction. In fact, berberine decreases citrate synthase activity in all the cell lines. Our results are in agreement with those of other authors that not only have found that berberine is localized in mitochondria^27^ but also that it is able to reduce oxygen-dependent glucose oxidation in these organelles in non-tumor cells such as myotubes and adipocytes^35^ and to reduce glucose, oxygen consumption and proliferation in hepatoma cells^36^. On the contrary, berberine supplementation reverts mitochondrial dysfunction induced by high fat diet and hyperglycemia in skeletal muscle in rat in part by increasing citrate synthase activity^14^.

Berberine enhances procaspase-3 cleavage, caspase-3 activity and/or expression in several tumor cells^3,30,37,38^. The activity of the enzyme is unmodified by berberine in HDF, increased in MIA PaCa-2 cells and reduced in U343 cells. Other authors have found increased cleaved caspase-3 by western blotting following berberine treatment in U251 and U87 glioblastoma cells^39^. Therefore, berberine modifies the activity of caspase-3 in a cell-specific manner. Indeed, it is known that caspase-3 can be processed and activated by the action of initiator caspases such as caspase-9 and caspase-8 and it is also regulated by posttranslational modifications^40^. The decrease in viability could also be due to a caspase-3-independent cell death, already described as an effect of berberine both in tumor and non-tumor colon cells^41^.

The crosstalk between autophagic and apoptotic signaling pathways is complex. Some natural products and chemotherapeutic drugs induce both pathways. In many cases autophagy inhibits apoptosis and vice versa. In HDF 50 μM berberine for 48 hours does not alter the transcription of *BECN1* and *LC3*, does not change the pattern of LC3 expression in immunohistochemistry experiments, and does not induce expression of LC3-II, while it induces senescence, in agreement with the idea that autophagy and senescence might be two mutually exclusive mechanisms^7^. On the contrary, in MIA PaCa-2 cells this treatment induces senescence, apoptosis (measured as activation of caspase-3) and autophagy. It is unclear at the moment whether the activation of these processes occurs simultaneously or autophagy is induced first as a defense mechanism in response to a cellular stress induced by berberine and it is followed later by induction of apoptosis. It is interesting to note that the population of MIA PaCa-2 cells is heterogeneous^15^, and it is composed of small and large cells that might respond to berberine in a different manner. Similarly, the natural alkaloid matrine induces both caspase-3-dependent apoptosis and autophagy in hepatocarcinoma cells; in these cells, Beclin-1 mediates the crosstalk between autophagy and apoptosis^42^. Berberine increases senescence and autophagy in the absence of caspase-3-dependent apoptosis in U343 cells. In these cells, autophagy could either provide macromolecules necessary to maintain the senescent cells alive or to induce cell death. In U343 cells and MIA PaCa-2 cells, damaged mitochondria might have been destroyed in lysosomes after fusion of autophagosomes, causing at least in part the observed decrease of citrate synthase.

This is the first study showing that berberine regulates the expression of *DAP1*, a negative regulator of autophagy^43^. It is interesting to note that *DAP1* is also involved in the regulation of cell migration and tumor invasiveness in breast cancer cells^19^. The exposure to the non-toxic 0.4 μM dose of berberine reduced the migration and invasion capability of MIA PaCa-2 cells. Whether the increase in *DAP1* transcription results in the control of autophagy and/or inhibition of migration is unclear at the moment and needs further investigation. Berberine inhibits cell migration and/or invasion in several tumor cell lines^3,10,44–47^. Since the CXCL12-CXCR4 axis plays an important role in promoting the invasion capability^20^, we wondered whether berberine altered the expression profile of *CXCR4*. *CXCR4* is not transcribed in control or berberine-treated U343 cells that are unable to migrate in presence or absence of berberine. Berberine does not alter migration in HDF^48^. In contrast, berberine reduced migration and dramatically downregulated *CXCR4* in MIA PaCa-2 cells suggesting that this receptor is a possible target of the inhibitory effect of berberine. Migration suppression associated with berberine-induced downregulation of *CXCR4* has been also found in MCF-7 cells^44^ and in KYSE-30 cells^45^. Besides the regulation of signal transduction pathways, the direct interaction of berberine with the cellular cytoskeleton may underlie its inhibitory effect on cell migration and cell proliferation. Recently, it has been demonstrated that berberine binds to VASP and inhibits actin polimerization^49^ and it also binds to a single site of tubulin, inhibiting microtubule assembly and promoting depolimerization^50^.

In summary, we have demonstrated that berberine induces cell cycle arrest in G2, senescence, but not autophagy in HDF. U343 cells are also arrested in G2, but berberine induces both senescence and autophagy, while in MIA PaCa-2 cells the alkaloid induces arrest in G1, senescence, autophagy, increase of caspase-3 activity and impairs migration. Moreover, the three cell lines show mitochondrial dysfunction as shown by decreased citrate synthase activity. The ability of berberine to induce cell cycle arrest, senescence and autophagy in U343 and MIA PaCa-2 cells and to impair migration in MIA PaCa-2 cells are interesting features that allow its potential use as an adjuvant in chemotherapy.

## Methods

### Cell culture and treatments

We used the human glioblastoma U343 GBM cells (U-343 MGa, CLS Cell Lines Service), the pancreatic carcinoma MIA PaCa-2 cells and normal primary dermal fibroblasts HDF (American Type Culture Collection ATCC, USA). The cells were cultured in Dulbecco’s modified Eagle’s medium (DMEM, Sigma) supplemented with 10% fetal bovine serum (Sigma), 100 U/ml penicillin and 100 mg/ml streptomycin (Gibco). All cells were maintained at 37 °C in humidified air with 5% CO_2_. Berberine dilutions were obtained from a 10^−2^ M berberine chloride (Sigma) stock solution prepared in DMSO (Sigma). Control cells were treated with corresponding volumes of DMSO. Every 24 hours the treatments were renewed.

### Confocal microscopy of berberine intracellular distribution

Cells were cultured in 12-well culture dishes and treated for 1 hour or 48 hours with 10 μM, 50 μM, 150 μM berberine or DMSO. After treatments, the *in vivo* cells were visualized by a LEICA TCS SP8 confocal laser scanning microscope (Leica Microsystems, Mannheim, Germany) equipped with a HC PL APO CS2 63x/1.40 oil objective and using a combined sequential procedure with fluorescence channels and differential interference contrast. The tetramethylrhodamine methyl ester (TMRM, Invitrogen) that was used as a tracer of mitochondria^14^ was added to cells 30 minutes before the end of treatments. Images of berberine and TMRM signals, acquired by laser Argon 488 and DPSS 561 with setting of Hybrid detectors (HyDs, Leica Microsystems, Mannheim, Germany) non-overlapping, 495–548 nm and 568–700 nm, respectively, were merged to visualize their co-localization in mitochondria. All confocal acquisitions images were taken at the same exposure times.

### Trypan blue exclusion cell counting assay

The cells were incubated for 48 hours with media containing 0.4 μM, 2 μM, 10 μM, 50 μM berberine or DMSO in T25 flasks. After 48 hours of treatments the cells were detached with trypsin-EDTA (Sigma) and suspended in 50% supplemented cell culture medium 50% Trypan blue (3*Z*,3′*Z*)-3,3′-[(3,3′-dimethylbiphenyl-4,4′-diyl)di(1*Z*)hydrazin-2-yl-1-ylidene]bis(5-amino-4-oxo-3,4-dihydronaphthalene-2,7-disulfonic acid) (Sigma). Cell count was performed using a Neubauer chamber.

### Citrate synthase assay

Cells were incubated with berberine for 48 hours, washed with PBS (Sigma), trypsinized and resuspended in 400 µl of a lysis buffer containing 100 mM Tris-HCl pH 7.4, protease inhibitors cocktail (Sigma), 10 mM NaF, 30 mM β-glycerophosphate and 10 mM sodium pyrophosphate as phosphatase inhibitors. Crude extracts were obtained by three freeze/thaw cycles followed by centrifugation at 10000xg for 40 min at 4 °C; supernatant was collected and protein content determined^51^. Citrate synthase (CS) assay was performed according to^52^ with some modifications. The assay was carried out by continuous spectrophotometric monitoring of the change in absorbance of 5,5′-Dithiobis(2-nitrobenzoic acid) (DTNB) at 412 nm at 30 °C (extinction coefficient of 13600 M^−1^cm^−1^). The enzyme assay mixture contained: 80 mM Tris-Cl pH 8.3, 0.5 mM acetyl-CoA, 5 mM oxaloacetate, 10 mM DTNB and 30 µg of crude extract.

### Cell Cycle Analysis

Cell cycle distributions were determined using propidium iodide (PI) (Sigma) staining and flow cytometry. After being treated with 10 μM or 50 μM berberine for 48 hours, cells were washed with PBS, trypsinized, fixed in 95% cold ethanol diluted 3:1 in PBS and stored at 4 °C overnight. Cell pellets were washed twice with PBS, resuspended in staining solution (50 µg/mL PI, 0.1% sodium citrate, 0.5 mg/mL RNase A and 0.1% Nonidet NP40) and incubated overnight at 4 °C. Cell cycle distributions were determined using flow cytometry (BD FACSJazz™ Cell Sorter, BD Bioscience) and data were analyzed using BD FACS Software (BD Bioscience). At least 10,000 cells were measured per sample.

### Analysis of Caspase-3 activity

After 48 hours of treatment with 50 μM berberine cells were detached with trypsin-EDTA (Sigma), and centrifuged. The pellet was washed in PBS and treated for 30 minutes at 4 °C with lysis buffer (Tris-HCl 20 mM pH 7.4, 150 mM NaCl, 1 mM DTT, 5 mM EDTA, 5 mM EGTA, 1% TRITON X-100, Protease Inhibitor Cocktail (Sigma). Lysates were separated by centrifugation at 10000 rpm for 15 minutes at 4 °C and the supernatant collected. Protein content was determined by Bradford method^51^, with bovine serum albumin as a standard. Determination of Caspase-3 activity was carried out in a 96-well plate in a total volume of 100 µl of a buffer containing Tris-HCl 50 mM pH 7.4, DTT 10 mM. Sixty-150 µg protein were incubated for 5 hours in presence of 200 μM Ac-DEVD-pNA Caspase-3 substrate (Enzo Life Sciences), and the released pNA was measured in the Ultra Microplate reader (Bio-Tek Instruments Inc.) at 405 nm every hour using KC Junior software.

### SA β-gal activity assay

SA β-gal activity was examined after treatment with 10 μM or 50 μM berberine for 48 hours. The cells were seeded in 6-well plates and 48 hours later fixed for 3–5 minutes at room temperature in PBS containing 2% formaldehyde + 0.2% glutaraldehyde. Cells were then washed three times with PBS and incubated at 37 °C with fresh SA β-gal staining solution containing 1 mg/ml X-Gal, 5 mM potassium ferrocyanide, 2 mM MgCl_2_ in PBS pH 6.0. After 48 hours the cells were washed with PBS and stained with 20μg/ml Hoechst 33342 for 10 minutes. Wells were examined with a Nikon Eclipse TE200 inverted fluorescence microscope to count SA β−gal-positive cells and Hoechst-positive nuclei. The ratio SA β-gal cells/Hoechst-positive nuclei was calculated in each independent experiment.

### Immunofluorescence experiment with LC3B

Cells were seeded onto cover-slides the day before starting experiments. After treatment as described above, cells were washed with PBS, prefixed and permeabilized in ice-cold 100% methanol for 10 min at −20 °C. Slides were air dried and washed with PBS before incubation for 1 hour at 37 °C with the LC3B rabbit primary antibody (Cell Signaling 1:200 in PBS/1% BSA/0.1% Tween20). Slides were washed three times with PBS/0.1% Tween20 and incubated for 1 hour at RT with the donkey anti-rabbit secondary antibody (Alexa Fluor® 568, abcam) diluted 1:1000 in PBS/1% BSA/0.1% Tween20. Slides were washed twice with PBS and counterstained with Hoechst 1:1000 in PBS. Images were taken on a LEICA TCS SP8 confocal laser scanning microscope (Leica Microsystems, Mannheim, Germany).

### Western blotting

Berberine-treated or untreated cells were washed with 1 ml cold PBS containing protease inhibitor cocktail, supplemented with 1 mM PMSF, 1 mM sodium orthovanadate, 5 mM sodium pyrophosphate, 20 mM β-glycerophosphate. Medium was then removed, cells scraped off and collected. Plates were then washed with 1 ml cold PBS containing protease and phosphatase inhibitors and remaining cells collected and added to the previous ones, then centrifuged at 700xg for 5 min at 4 °C. Supernatants were discarded and 200 µl of lysis buffer containing 150 mM NaCl, 50 mM NaF, 0.5 mM EDTA pH 8.0, 0.5% Triton X-100, 1 mM PMSF, 1 mM sodium orthovanadate, 5 mM sodium pyrophosphate, 20 mM β-glycerophosphate in 25 mM Tris-Cl pH 7.4, was added. Vials with cells and lysis buffer were then strongly shacked for 1 min and kept on ice for 10 min prior to centrifugation at 10000xg for 40 min at 4 °C. Cellular lysates were collected and concentration of protein extracts was determined. Protein samples (30 µg each lane) were resolved by 15% SDS-PAGE at 200 V for 45 min and transferred onto polyvinylidene fluoride (PVDF) membranes at 90 V for 1 hour using Bio-Rad transfer system. Membranes were blocked with TBST (TBS supplemented with 0.1% (v/v) Tween-20) containing 5% (w/v) skim milk powder. Membranes were incubated with primary antibody overnight at 4 °C and with secondary antibody for 1 hour at room temperature before visualizing chemiluminescence of protein bands using Chemiluminescence Detection System. Primary antibodies specific for LC3 (Cell signaling, 1:1000 in TBST), actin (Novus Biological 1:20000 in TBST containing 3% (w/v) skim milk powder), and HRP-linked secondary antibodies anti-mouse and anti-rabbit IgG (Cell signaling) 1:3000 in TBST containing 5% skim milk were used.

### Wound healing assay

U343 and MIA PaCa-2 cells were seeded in 24-well culture dishes. When cells were confluent, scratches were made with a 200 μl sterile pipette tip, cellular debris was removed by gentle washing in culture medium. Thereafter, medium was replaced by DMEM containing different berberine concentrations (0.4 μM, 2 μM, 10 μM or DMSO) and the cells were allowed to migrate for 24, 48 or 72 hours. The photos of the distance between the wound edges were taken from the same regions under a Nikon Eclipse TE200 inverted optical microscope at 0, 24, 48 or 72 hours. The gap size was analyzed using Image-J 1.50 software.

### Cell invasion assay

The effect of berberine on the invasion capability of MIA PaCa-2 cells was determined using Cytoselect Cell invasion assay kit, basement membrane (Cell Biolabs). About 300,000 cells in serum-free DMEM containing 0.4 μM, 2 μM, 10 μM berberine or DMSO were seeded onto the upper trans-well chamber of inserts according to the manufacturer’s guidelines. Serum-containing DMEM was added to the lower chambers. After 48 hours of incubation, filter inserts were removed from the wells. After staining, the invaded cells were counted under a Nikon Eclipse TE200 inverted optical microscope. To rule out the possibility that the decreased cell invasion might be caused by declined total cell number, we performed a WST assay (Roche) by plating cells at the same density as into trans-well chambers for invasion assay. The relative invasive score was calculated as number of berberine-treated invaded cells (normalized versus DMSO group)/total cell number (measured by lecture at 570 nm of berberine-treated cells and normalized versus DMSO group*)*.

### RNA extraction, cDNA synthesis and Real Time RT-PCR

Cells were treated with 10 μM, 50 μM, 150 μM berberine or DMSO for 1, 24 or 48 hours. RNA was isolated by using EuroGold total RNA kit (Euroclone). The quantity and purity of RNA was assessed using the Biophotometer D30 Eppendorf. Before cDNA synthesis, RNA samples were tested for the absence of genomic DNA. cDNA was synthesized using Quanti tectH reverse transcription kit (Qiagen). Real-time RT-PCRs were performed with the Rotor Gene 6000 (Corbett Research, Explera), using SYBR Green as fluorescent dye, according to the MIQE guidelines^53^. The thermal cycler conditions were: 40 cycles of 95 °C for 15 seconds and 60 °C for 30 seconds. Preliminary experiments were performed to evaluate the transcriptional stability of candidate endogenous reference genes^54^. *Actin-β* was chosen by Normfinder Software^55^ as the most stable among the following housekeeping genes: *actin-β* (*ACTB)*, *Hypoxanthine Phosphoribosyltransferase 1 (HPRT1)*, *TATA Box Binding Protein* (*TBP)*, *Glyceraldehyde-3-Phosphate Dehydrogenase* (*GAPDH)*. We analyzed the gene expression levels of: *P53, P21, P16, BECN1, LC3, DNMT1, DNMT3A, DNMT3B, MGMT*, and *CXCR4* and *DAP1*. Primer sequences are available on request. Relative mRNA expression (fold change) was calculated with the Rotor Gene 6000 software v1.7 using Livak&Schittgen CT comparative model^56^.

### Statistical analysis

Data are expressed as mean ± S.D. of at least three independent experiments with at least three replicates. Data tabulation and descriptive statistics were performed by using Excel program (Office 2016). Statgraphics software (version XVI) was used for statistical analyses. Normality was tested by Shapiro-Wilk and Kolmogorov-Smirnov tests. Homoscedasticity was tested by Levene’s test. For multiple comparisons of normally distributed data, one-way ANOVA analysis of variance with the Tukey’s HSD post-hoc test was performed. For two independent samples comparison t-test was performed. P-values < 0.05 were considered to be statistically significant.

### Data availability

The datasets generated during and/or analysed during the current study are available from the corresponding author on reasonable request.

## Electronic supplementary material

Supplementary Figure SI1
