# Supplementary material for: Cell-specific pattern of berberine pleiotropic effects on different human cell lines
Source: Sci Rep. 2018 Jul 13;8:10599. doi: 10.1038/s41598-018-28952-3 (PMC6045596; doi:10.1038/s41598-018-28952-3)
Supplement: Supplementary file 1 — Supplementary Figure SI1 [file 41598_2018_28952_MOESM1_ESM.pdf]

Cell-specific pattern of berberine pleiotropic effects on different human cell lines

Alessandro Agnarelli, Marco Natali, Mercedes Garcia-Gil, Rossana Pesi,
Maria Grazia Tozzi, Chiara Ippolito, Nunzia Bernardini, Robert Vignali, Renata Batistoni,
Anna Maria Bianucci, Silvia Marracci

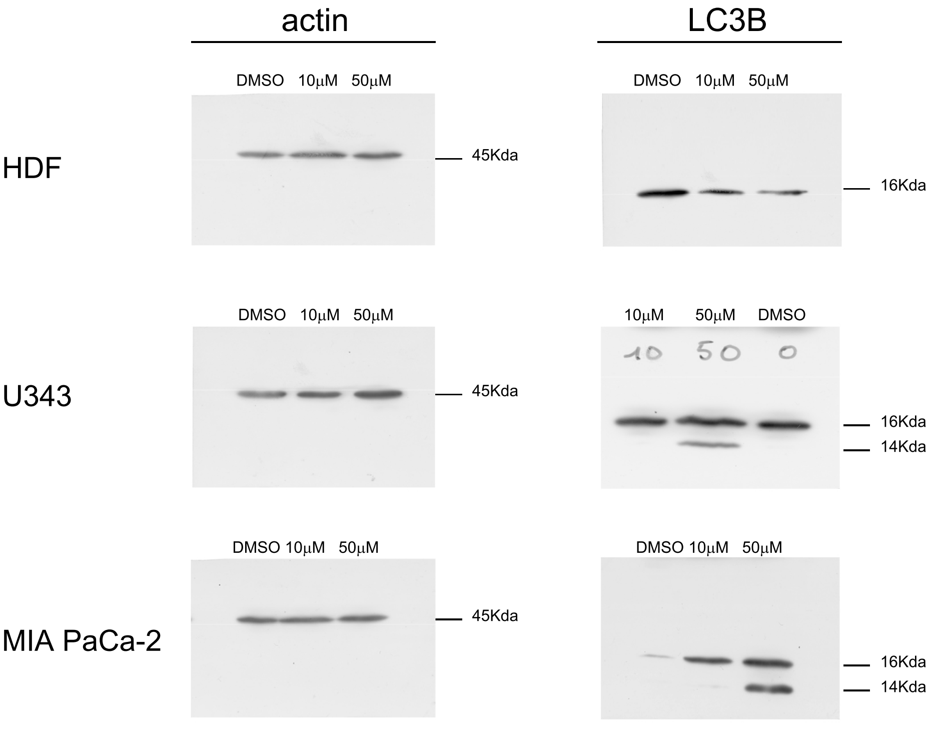

Western Blotting experiments. Cells are treated with DMSO or berberine for 48 hours and subjected to electrophoresis. Two gels were loaded with the same amount of proteins and run simultanously. Western Blotting was performed, as described in Methods, with actin or LC3B antibody. For the construction of Fig.6, in U343 (LC3B), lane 0 berberine was cropped on the right of the figure and pasted on the left in order to obtain the same sequence of the other figures (0, 10, 50 μM berberine).
